# Cobb syndrome: an interdisciplinary approach between neurosurgery and dermatology – case report and review of the literature

**DOI:** 10.1093/jscr/rjaf411

**Published:** 2025-06-27

**Authors:** Jason Riveros-Ruiz, Nory Huancahuari, Lesdy M Taipe-Huamani, Milagros Cairapoma

**Affiliations:** Department of Neurosurgery, Hospital Nacional Daniel Alcides Carrión, Av. Elmer Faucett s/n, Callao Province, Callao Region, Perú; School of Medicine, Universidad Nacional de San Cristóbal de Huamanga, Portal Independencia 57, Plaza de Armas, Huamanga District, Ayacucho Region, Perú; School of Medicine, Universidad Nacional de San Cristóbal de Huamanga, Portal Independencia 57, Plaza de Armas, Huamanga District, Ayacucho Region, Perú; Department of Dermatology, Hospital Nacional Daniel Alcides Carrión, Av. Elmer Faucett s/n, Callao Province, Callao Region, Perú

**Keywords:** Cobb syndrome, spinal vascular malformation, neurocutaneous disorders

## Abstract

Cobb syndrome is rare in the international literature. It is characterized by segmental cutaneous and spinal vascular malformations within the same metamere. We present the first documented case in Peru. We present a case of a patient with paraparesia and congenital violaceous-red cutaneous patch in a metameric distribution. Lumbar spine magnetic resonance imaging revealed spinal canal involvement at T8 level, prompting decompressive laminectomy and lesion biopsy. Histopathological analysis demonstrated dermal capillary proliferation and mild polymorphonuclear infiltrate. Adjuvant treatment was initiated. At 9-month follow-up, significant improvement in bilateral lower limb motor strength was noted. The identification of segmental lesions, combined with clinical correlation and biopsy-confirmed spinal vascular malformation, was critical in differentiating Cobb syndrome from other neurocutaneous disorders. This case underscores the importance of interdisciplinary collaboration in diagnosing and managing rare neurocutaneous syndromes.

## Introduction

Cobb syndrome, alternatively termed spinal arteriovenous metameric syndrome (SAMS) or cutaneous meningospinal angiomatosis, was first reported in 1890 and later characterized by Stanley Cobb in 1915, establishing its eponym [1]. This exceptionally rare neurocutaneous disorder is defined by the coexistence of vascular malformations affecting both the skin and spinal cord within identical metameric (somite-derived) segments [2]. The pathognomonic association between cutaneous and neural lesions stems from their shared embryological origin in ectodermal derivatives, which gives rise to concurrent involvement of these seemingly disparate tissues [3, 4].

A principal diagnostic dilemma in adult patients lies in the frequent asymptomatic latency period, during which pathognomonic cutaneous markers may remain the sole manifestation for years prior to neurological deterioration [5]. Nevertheless, early identification of these dermatological signs is imperative, as they may herald clinically silent spinal vascular anomalies capable of precipitating irreversible myelopathic deficits [4]. The inherent complexity of Cobb syndrome necessitates a rigorously coordinated, multidisciplinary therapeutic strategy.

With fewer than 150 cases documented globally [6] and no prior reports in Peruvian medical literature, we present a seminal case of an adult patient exhibiting progressive paraparesia with metameric cutaneous lesions. This case exemplifies the diagnostic challenges and underscores the efficacy of an integrated neurosurgical-dermatological approach in achieving favorable outcomes.

## Case report

**Figure 1:**
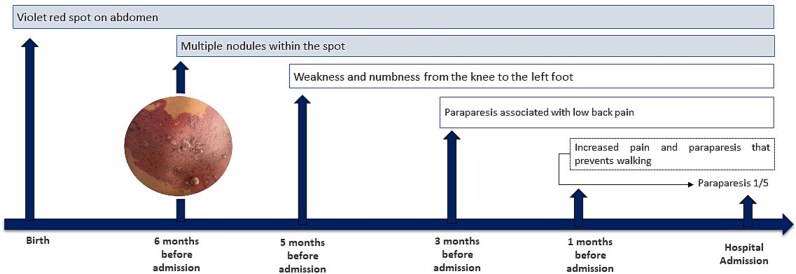
Clinical progression from congenital cutaneous manifestations to hospital admission. AVM (arteriovenous malformation), MRI (magnetic resonance imaging), MRA (magnetic resonance angiography), MRV (magnetic resonance venography), CT (computed tomography).

A 24-year-old male merchant from Northern Peru with no significant medical history, who has provided informed consent for this report to be published, reported the presence of a congenital violaceous-red cutaneous patch on his abdomen. Six months prior to admission, he noted development of a nodular lesion within this pre-existing dermatological marking. His neurological symptoms progressed sequentially: initial left lower extremity paresis developed 5 months pre-admission, followed 3 months later by paraparesia accompanied by non-radiating lumbar pain (Fig. 1).

Dermatoscopic examination demonstrated a pink-red background with linearly distributed vascular structures throughout the lesion, accompanied by multiple violaceous-red lacunae interspersed with dotted and globular features. The nodular component exhibited reddish lacunae with central yellowish scarring. Magnetic resonance imaging revealed an infiltrative process replacing normal bone marrow architecture from T8 to L1 vertebral levels, displaying mixed hypo- and hyperintense signals on T1/T2-weighted sequences with homogeneous contrast enhancement and epidural extension.

**Figure 2:**
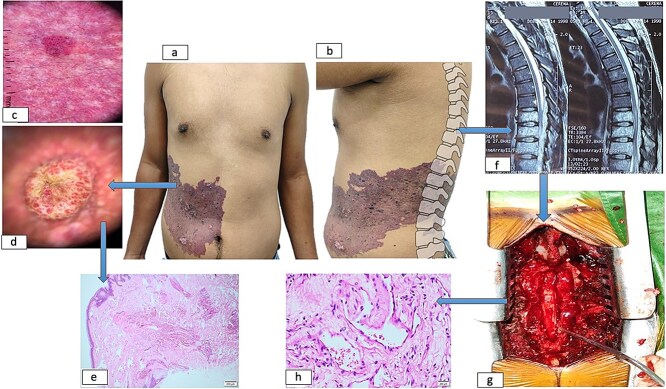
Dermatological and spinal manifestations of cobb syndrome. (a–b) Metamerically distributed cutaneous lesions observed in frontal (a) and sagittal (b) views. (c) Dermoscopic examination reveals a background with linearly distributed vascular structures throughout the lesion, accompanied by lacunae containing scattered dots and globules. (d) Nodular component demonstrates lacunae with central scarring. (e) Histopathological analysis of skin biopsy shows proliferation of capillaries and medium-caliber venous vessels in superficial and mid-dermis. (f) Spinal imaging reveals vertebral and medullary infiltration at thoracic levels. (g) Postoperative spinal column following decompressive laminectomy. (h) Vertebral biopsy confirms vascular infiltration.

The patient underwent decompressive laminectomy at T7-T9 levels, with concurrent vertebral body biopsy revealing histopathological evidence of vascular infiltration consistent with angiomatous lesions and lamellar bone and fibrous connective tissue with prominent vascular ectasia. Complementary cutaneous histopathological analysis demonstrated nodular proliferation of venous vascular channels (Fig. 2).

A consensus diagnosis of Cobb syndrome was established through interdisciplinary consultation involving neurosurgery, dermatology, pathology, and radiology teams. This integrated assessment confirmed metameric alignment between cutaneous and spinal vascular malformations. Adjuvant corticosteroid therapy was initiated for perilesional medullary edema control. At 12-month follow-up, the patient maintained a preserved motor function (5/5 strength throughout) and a no emergence of new dermatological lesions.

## Discussion

Neurocutaneous syndromes frequently serve as sentinel manifestations of underlying neurological pathology, with external cutaneous abnormalities providing critical diagnostic clues to concurrent involvement of both the nervous and integumentary systems [7, 8]. Given their inherently multisystemic nature, these conditions demand a comprehensive multidisciplinary approach to patient management [9].

In the present case, the identification of vertebral vascular infiltration suggestive of hemangioma, when coupled with progressive neurological deficits and characteristic dermatological lesions, necessitated consideration of broader pathological associations [2, 10]. Pediatric and young adult patients presenting with segmental vascular malformations warrant thorough evaluation including selective spinal angiography or magnetic resonance angiography to exclude spinal cord vascular anomalies, complemented by histopathological examination to establish definitive diagnosis. The microscopic similarities between Cobb syndrome and Klippel-Trénaunay-Weber syndrome underscore the importance of clinical correlation, as neurological involvement remains pathognomonic for Cobb syndrome [11]. Distinctively, Sturge–Weber syndrome manifests with cognitive impairment, motor dysfunction, and capillary malformations, with glaucoma representing a hallmark feature [12, 13]. The differential diagnosis must carefully consider neurofibromatosis type 1 (characterized by café-au-lait macules), tuberous sclerosis (with its rare spinal involvement and hypomelanotic macules), and metastatic disease (typically presenting with vertebral destruction in the absence of cutaneous lesions) [14] (Table 1).

**Table 1:** Comparative clinical and pathological features of cobb syndrome and major neurocutaneous/vascular differential diagnoses.

**Feature**	**Cobb Syndrome**	**Klippel-Trénaunay-Weber**	**Sturge–Weber syndrome**	**Neurofibromatosis type 1**	**Vertebral metastases**	**Tuberous sclerosis**
**Key characteristics**	Metameric spinal AVMs + cutaneous vascular lesions	Capillary/ venous/ lymphatic malformations+ limb hypertrophy	Facial port- wine stain + leptomeningeal angiomatosis	Café-au-lait spots, neurofibromas, Lisch nodules	Osteolytic/ blastic lesions without cutaneous correlation	Facial angiofibromas, shagreen patches, cortical tubers
**Spinal involvement**	Vascular malformations (AVMs, hemangiomas) in same metamere as skin lesions	Rare spinal AVMs; mainly paraspinal vascular anomalies	Rare; if present, leptomeningeal angiomatosis may extend tox spine	Plexiform neurofibromas may compress spine	Vertebral destruction (compression fractures, epidural mass)	Rare; possible subependymal nodules in central canal
**Cutaneous findings**	Violaceous patch/angioma in dermatomal distribution	Port-wine stain, venous varicosities, lymphatic malformations	Unilateral port- wine stain (V1- V2 trigeminal distribution)	≥6 café-au-lait spots (>5 mm prepubertal, >15 mm postpubertal), axillary freckling	Usually absent (except in rare cutaneous metastases)	Hypomelanotic macules, facial angiofibromas, periungual fibromas
**Imaging findings**	Spinal AVM/hemangioma on MRI/MRA; vertebral angiomatosis	Soft-tissue hypertrophy, venous malformations on MRI/MRV	Cortical calcifications (‘tram-track’), pial angiomatosis on MRI	Plexiform neurofibromas, optic pathway gliomas	Osteolytic/blastic lesions on CT; epidural mass on MRI	Cortical tubers, subependymal nodules on MRI
**Histopathology**	Proliferation of abnormal blood vessels (dermis + spinal biopsy)	Dilated capillaries/venules in dermis; lymphatic anomalies	Thin-walled vascular channels in leptomeninges	Neurofibroma: spindle cells in collagenous matrix	Tumor cells consistent with primary malignancy (e.g. adenocarcinoma)	Cortical tubers: disorganized neurons/glia; subependymal giant cell astrocytomas
**Genetic basis**	Sporadic (no known gene)	PIK3CA mutations (somatic mosaicism)	GNAQ mutations (somatic mosaicism)	NF1 gene mutations	None (secondary to systemic cancer)	TSC1/TSC2 mutations
**Neurologic symptoms**	Myelopathy (paraparesis, sensory deficits) due to spinal AVM compression/bleeding	Limb asymmetry, chronic pain	Seizures, contralateral hemiparesis, developmental delay	Learning disabilities, optic glioma-related vision loss	Local pain, pathologic fractures, cord compression	Epilepsy, intellectual disability, autism spectrum disorder

Therapeutic intervention in our Cobb syndrome case prioritized neurological stabilization through decompressive laminectomy, as progressive sensorimotor deficits indicated imminent neurological compromise [15]. The essential role of multidisciplinary collaboration is evidenced by the demonstrated efficacy of corticosteroids for symptomatic management [1], while emerging therapies such as the kinase inhibitor trametinib show promise for genetically associated phenotypes [16].

In conclusion, timely diagnosis through integrated clinical, radiological, and histopathological evaluation proves critical for distinguishing neurocutaneous disorders and implementing targeted therapeutic strategies. The prevention of disease progression remains the fundamental objective of clinical management in these complex cases. Our experience emphasizes the value of systematic evaluation incorporating advanced imaging modalities and histopathological analysis to guide appropriate intervention and optimize patient outcomes.
